# Perspectives on Stem Cell Therapy in Diabetic Neuropathic Pain

**DOI:** 10.3390/neurolint16050070

**Published:** 2024-08-30

**Authors:** Tadeu Lima Montagnoli, Aimeé Diogenes Santos, Susumu Zapata Sudo, Fernanda Gubert, Juliana Ferreira Vasques, Rosalia Mendez-Otero, Mauro Paes Leme de Sá, Gisele Zapata-Sudo

**Affiliations:** 1 Programa de Pós-Graduação em Farmacologia e Química Medicinal, Instituto de Ciências Biomédicas, Universidade Federal do Rio de Janeiro, Rio de Janeiro 21941-902, Brazil; tmontagnoli@gmail.com (T.L.M.); aimeediogenessantos@gmail.com (A.D.S.); 2 Programa de Pós-Graduação em Medicina (Cirurgia), Faculdade de Medicina, Universidade Federal do Rio de Janeiro, Rio de Janeiro 21941-902, Brazil; susumu_sudo@hotmail.com (S.Z.S.); paesleme@hucff.ufrj.br (M.P.L.d.S.); 3 Instituto de Ciências Biomédicas, Universidade Federal do Rio de Janeiro, Rio de Janeiro 21941-902, Brazil; fernanda.gubert@icb.ufrj.br; 4 Instituto de Biofísica Carlos Chagas Filho, Universidade Federal do Rio de Janeiro, Rio de Janeiro 21941-902, Brazil; julianavasques@biof.ufrj.br (J.F.V.); rmotero@biof.ufrj.br (R.M.-O.); 5 Instituto do Coração Edson Saad, Faculdade de Medicina, Universidade Federal do Rio de Janeiro, Rio de Janeiro 21941-902, Brazil

**Keywords:** diabetes, neuropathic pain, stem cells, neuroinflammation, glial activation

## Abstract

Diabetes mellitus-related morbidity and mortality are primarily caused by long-term complications such as retinopathy, nephropathy, cardiomyopathy, and neuropathy. Diabetic neuropathy (DN) involves the progressive degeneration of axons and nerve fibers due to chronic exposure to hyperglycemia. This metabolic disturbance leads to excessive activation of the glycolytic pathway, inducing oxidative stress and mitochondrial dysfunction, ultimately resulting in nerve damage. There is no specific treatment for painful DN, and new approaches should aim not only to relieve pain but also to prevent oxidative stress and reduce inflammation. Given that existing therapies for painful DN are not effective for diabetic patients, mesenchymal stromal cells (MSCs)-based therapy shows promise for providing immunomodulatory and paracrine regulatory functions. MSCs from various sources can improve neuronal dysfunction associated with DN. Transplantation of MSCs has led to a reduction in hyperalgesia and allodynia, along with the recovery of nerve function in diabetic rats. While the pathogenesis of diabetic neuropathic pain is complex, clinical trials have demonstrated the importance of MSCs in modulating the immune response in diabetic patients. MSCs reduce the levels of inflammatory factors and increase anti-inflammatory cytokines, thereby interfering with the progression of DM. Further investigation is necessary to ensure the safety and efficacy of MSCs in preventing or treating neuropathic pain in diabetic patients.

## 1. Introduction

Increased prevalence of diabetes mellitus (DM) worldwide is of great concern in an aged population because of the occurrence of long-term complications such as retinopathy, nephropathy, cardiomyopathy, and neuropathy. The incidence of neuropathy in diabetic patients is approximately 20%, and this chronic complication is of particular importance when the mortality rate is 27% over ten years [1]. Diabetic neuropathy (DN) is one of the main complications that arises with the chronic evolution of DM, which is characterized by the progressive degeneration of axons and nerve fibers due to chronic exposure to hyperglycemia. Its most common presentation is distal and symmetrical polyneuropathy with increased sensitivity to pressure and temperature. Painful DN or diabetic neuropathic pain is a major source of morbidity with hyperalgesia (increased response to painful stimuli) and allodynia (response to painless stimuli), and the progression of the disease can develop into hypoalgesia through sensory and motor nerve dysfunction. 

The pathogenesis of DN is complex and poorly understood, but it is primarily associated with oxidative stress, neuroinflammation, and endoneural hypoxia, all exacerbated by hyperglycemia [2]. Mechanisms involve not only neurons but also supporting glia (Schwann cells in peripheral nerves; satellite glial cells in dorsal root ganglia, DRG; astrocytes and microglia in the spinal cord), components of the immune and inflammatory system, and neurotrophic factors [3]. Reactive species generated from hexose and lipid metabolism could be involved in nerve inflammation and contribute to neurovascular damage, leading to axonal degeneration and endothelial dysfunction [4]. Current recommendations for the treatment of neuropathic pain achieve 30% pain reduction in almost 30% of cases and are limited to analgesia, despite the proven involvement of glycotoxicity to the nervous system. Research into new alternative therapeutic interventions is important since the ideal regimen remains undefined. 

Mesenchymal stromal cells (MSCs)-based therapies represent a promising approach, particularly due to their well-documented anti-inflammatory, angiogenic, and neuroprotective potential [5]. MSCs can be isolated from adult or perinatal tissues, including bone marrow (BM), adipose tissue (AT), peripheral blood, and umbilical cord (UC), each with specific characteristics and properties. After transplantation, fewer than 1% of MSCs reach the injured tissue, with most cells remaining in liver, spleen, and lungs for a few days [6]. Initially, MSCs were proposed as a treatment based on their proliferative potential and regenerative capacity. However, it was recently demonstrated that MSCs also exert anti-inflammatory and immunomodulatory responses [7]. Moreover, MSCs secrete cytokines and growth factors, which counteract apoptosis and promote regeneration and neovascularization [8], and these functions have been evaluated both in animal models and clinical trials [9]. Although BM-derived MSCs were originally used for demonstration of their clinical indications, UC has become the preferred source due to the invasive nature of isolation from BM [10]. MSC therapy has shown efficacy in treating DM, as UC-MSCs play a reparative role by stimulating islet beta-cell regeneration and improving pancreas function [11]. Additionally, MSCs also reduce immune system activation, limit pancreas degeneration, and shift the balance between pro-inflammatory and anti-inflammatory factors, thereby decreasing apoptosis and inflammation [12]. The angiogenic properties of MSCs induce vascular repair, aiding in the recovery of peripheral vascular disease and cutaneous wounds. Therefore, patients in the early stages of DM may particularly benefit from MSC treatment [13]. This review summarizes both preclinical and clinical evidence in support of the importance of regenerative medicine using MSCs for the treatment of diabetic neuropathic pain. 

## 2. Diabetic Neuropathy and Diabetic Neuropathic Pain

DN affects about 50% of all diagnosed DM cases, and symptoms depend on the affected nerve. In the early stage, this complication. characterized by damage to peripheral nerves, first affects the poorly myelinated sensory and autonomic neurons. In contrast, fully myelinated motor nerve axons display some degree of resistance to metabolic changes, as motor alterations are observed only in long-term disease [14]. Nerve injury is characteristic of DN, with reduction in conduction velocity in both sensory and motor fibers [15]. DM-induced autonomic neuropathy results in dysfunction of sympathetic and parasympathetic nervous systems, leading to symptoms such as syncope, exercise intolerance (cardiac), nausea, vomiting (gastrointestinal), and impotence (sexual).

The pathogenesis of DN is complex and involves factors such as hyperglycemia, nerve ischemia, and demyelination [16]. Metabolic disturbances caused by increased plasma glucose levels result in excessive activation of the glycolytic pathway in nerves and Schwann cells. This leads to increased formation of lactate and pyruvate and activate pentose phosphate and polyol pathways, provoking oxidative stress and mitochondrial dysfunction, which ultimately damage the nerves [17]. Dyslipidemia also contributes to the progression of DN since the increase in triglycerides and cholesterol levels causes neuronal damage. Oxidized or glycated low-density lipoprotein bind to neuronal receptors, inducing inflammatory conditions and increasing the production of reactive oxygen species (ROS) [18]. Glycemic variability is a significant factor in the development of micro- and macrovascular alterations [19]. Initially, microvascular complications were thought to be the primary contributors to DN [20], driven by nitric oxide deficiency and the enhanced activity of free radicals [21]. Randomized clinical trials showed that glycemic control slows the progression of microvascular complications of DM [22] but without reduction of DN symptoms [16]. Advanced glycation end-products (AGEs) also exacerbate DN by inducing vasoconstriction and potentiating vascular injury. Activation of AGE receptor (RAGE) stimulates the nuclear factor kappa-light-chain enhancer of activated B cells (NF-KB), which, in turn, regulates inflammation and apoptosis [21]. Intense local inflammation is currently considered a major condition for nerve damage [23]. Increased inflammatory gene expression in macrophages observed in patients with DN reinforce the importance of nerve inflammation to pathogenesis [24]. 

Oxidative stress plays a significant role in DN pathogenesis due to the accumulation of free radicals and the reduced activity of antioxidant enzymes [25]. Mitochondrial injury induced by DM uncouples ATP synthesis and promotes generation of ROS [26]. Studies evaluating the use of antioxidants such as lipoic acid to prevent DN in rats demonstrated increased nerve blood flow, thereby slowing the progression of DN and a reduction in nerve damage [27]. The peripheral vasculopathy associated with DN may also lead to infected skin lesions and the evolution of tissue necrosis, obviating the need for amputations. This condition reaffirms the inflammatory component of DM, characterized by elevated cytokine levels, such as interleukin (IL)-1β, IL-6, and tumor necrosis factor (TNF)-α. Additionally, activated microglia in the spinal cord can also release pro-inflammatory cytokines, which play a crucial role in the development of hyperalgesia and allodynia [28]. 

Neuropathic pain is a symptom of DN and affects 15 to 25% of diabetic patients, often leading to a significant reduction in quality of life. Neuropathic pain arises as a consequence of lesions of the somatosensory nervous system, resulting in hyperalgesia and allodynia [29]. DM-induced peripheral nerve injury results in the hyperexcitability of A and C nerve fibers, which ultimately generates spontaneous action potentials towards the spinal cord. These changes are associated with the overexpression of voltage-gated sodium channels Na_v_1.7, 1.8, and 1.9 in C-fibers, along with increased intracellular calcium concentration [30]. Pain transmission is driven by the release of excitatory neurotransmitters in presynaptic terminals, particularly glutamate and substance P, which depolarize postsynaptic fibers. Substance P is mainly responsible for prolonging pain responses by stimulating mast cells to release histamine, which further reduces the excitation threshold of nociceptors. 

Once the pain signal reaches the cerebral cortex via supraspinal projections, it activates descending pathways that modulate pain sensation through the release of endorphins, enkephalins, serotonin, and noradrenaline. However, in diabetic neuropathic pain, this inhibitory mechanism is impaired, with reduced activation of interneurons leading to lower release of inhibitory neurotransmitters, such as γ-aminobutyric acid (GABA), glycine, enkephalins, endocannabinoids, and adenosine [31]. The exacerbated release of excitatory neurotransmitters, resulting from the disbalance of greater pain transmission and less activation of inhibitory pathways, leads to neuron excitotoxicity and activates glial cells, especially in the spinal dorsal horn [32,33]. Hyperglycemia can also activate mitogen-activated protein kinases (MAPKs), contributing to the development of painful DN, and their inhibition could reduce inflammation and pain [34]. 

## 3. Mesenchymal Stromal Cells as Alternative Therapy for Diabetic Neuropathy

In recent years, several studies have reported that treatment with MSCs from different tissues can improve neuronal dysfunction associated with DN. Intramuscular injection of BM-MSCs has been shown to restore the reduced motor (MNCV) and sensory (SNCV) nerve conduction velocities observed in DN in streptozotocin (STZ)–DM models. Accordingly, this therapy also increases mRNA levels of several angiogenic, pro-myelination, and neurotrophic factors, such as vascular endothelial growth factor (VEGF), myelin basic protein (MBP), and insulin-like growth factor (IGF)-1 [35]. Moreover, treatment with BM-MSCs also seems to regulate inflammation in the dorsal horn of the spinal cord. Intravenous transplantation of BM-MSCs in a STZ-DM mice model significantly reduced the astrogliosis and microgliosis observed in the spinal region, along with a decrease in inflammation, as evidenced by increased IL-10 and transforming growth factor (TGF)-β and reduced IL-1β and TNF-α levels [36]. BM-MSCs may also regulate blood glucose levels, reduce demyelination and the production of ROS, and enhance the expression of nerve growth factor (NGF) and VEGF in STZ-induced DN in rats. Additionally, BM-MSC can improve Schwann cell viability by modulating the GSK-3β/β–catenin pathway [37]. 

A transitory positive effect of BM-MSC therapy was observed after administration in the hind limbs, where, provisionally, it improved the sciatic–tibial MNCV, which was associated with a transient increase in the expression of the neurotrophic factors NGF and neurotrophin (NT)-3 [38]. Co-transplantation of BM-MSCs with pancreatic islets has been shown to increase the survival of transplanted cells while decreasing the dose required for glycemic control in animal models to only one-third of the standard islet cell dose. Another strategy by which to improve MSCs’ therapeutic potential is to precondition cell cultures with modulating factors before transplantation. Priming human BM-MSCs with toll-like receptor (TLR)-3 agonists result in a cell line with increased anti-inflammatory profile, referred to as “MSC2”. Intraperitoneal therapy with MSC2 in STZ-mice produced a greater beneficial effect on nociception thresholds compared to non-primed BM-MSCs. MSC2 therapy also reduced plasma levels of inflammatory factors IL-1α, IL-1β, and interferon-gamma (IFN-γ), while upregulating anti-inflammatory molecules [39]. The neurotrophic potential of BM-MSCs was further increased by preconditioning with fluoxetine. Intramuscular injection of fluoxetine-preconditioned BM-MSCs in diabetic rats provided protection of sciatic nerve fibers, along with an increase in brain-derived neurotrophic factor (BDNF), VEGF, and IL-10 mRNA levels [40]. 

When using dental-pulp-derived MSCs (DP-MSCs), it was demonstrated that repeated intramuscular injections were necessary to achieve long-lasting functional improvement [41]. Recovery of MNCV, improvement in nerve blood flow and integrity, and reduction in inflammation were identified as possible mechanisms [42,43]. In a similar way, intramuscular injection of MSCs isolated from adipose tissue (AT-MSCs) improved electromyography parameters and reduced the number of degenerated and demyelinated fibers in the sciatic nerve in STZ-DM mice. An increase in S100 and cyclin-dependent kinase (Cdk)-2—markers of Schwann cell proliferation and migration, respectively—was also detected, suggesting peripheral nerve regeneration [44]. AT-MSCs, pre-conditioned with the iron chelator deferoxamine (DFX), were found to stimulate the release of pro-angiogenic and anti-inflammatory molecules [45]. 

MSCs can also be genetically engineered to express specific factors of interest. For example, in in vitro models of direct and indirect co-culture, rat AT-MSCs were induced to overexpress erythropoietin, a hematopoietic cytokine involved in neuroprotection. Compared to their naïve counterparts, modified AT-MSCs enhanced cell viability and induced a greater reduction of ROS, such as superoxide, as well as apoptotic markers in sciatic nerves from STZ-DM rats [46]. 

Another source of MSCs is perinatal tissue, which shows increased therapeutic efficacy in angiogenesis models [47]. Intralimb injections of placenta-derived MSCs (P-MSCs) in diabetic mice did not alter blood glucose or body weight but did improve features of DN through stimulation of the Wnt signaling pathway in the nerves. This assumption was supported by the observation that the demyelination repair provided by P-MSCs was antagonized when a Wnt inhibitor was used [48]. In an attempt to improve the anti-inflammatory potential of umbilical-cord-derived MSCs (UC-MSCs), preconditioning with IFN-γ resulted in the inhibition of apoptosis in the sciatic nerve, as evidenced by a significant reduction in pro-apoptotic markers, caspase-3, and Bax, as well as intense upregulation of the anti-apoptotic factor Bcl-2 [49]. A combined therapy using UC-MSCs and resveratrol, a polyphenol with antioxidative and anti-inflammatory properties, also demonstrated promising cumulative benefits compared to isolated treatments. Histological analysis of the sciatic nerve from diabetic mice treated with the combination of UC-MSCs and resveratrol demonstrated better fiber alignment, reduced vacuolar degeneration of myelin, and increased the presence of new capillaries [50]. The paracrine effect of MSCs has also been tested, in which the intravenous administration of conditioned medium (CM) from human UC-MSCs protected the viability and angiogenic properties of greatly glucose-damaged cells [51].

Currently, cell-free therapy is the main focus of research, with the challenge of identifying the components in CM responsible for the therapeutic effects of MSCs. As a result, MSC-derived exosomes have been isolated and tested in DN. Exosomes are extracellular vesicles, 40–150 nm in diameter, originating from the endosomal pathway and enriched in promising therapeutic candidate molecules: microRNAs (miRNA) [52]. In DN models, intravenous injection of mouse BM-MSCs-derived exosomes (BM-exosomes) did not alter glucose metabolism but did regulate sciatic nerve inflammation by reducing macrophage number and promoting their polarization into an anti-inflammatory phenotype (M2). Along with modulation of inflammatory cells, BM-exosomes enhanced blood flow, leading to an improvement in the microvascular dysfunction observed in DN [8]. Recently, engineering techniques were employed to load BM-exosomes with miR-146a, an anti-inflammatory molecule related to DN pathogenesis. This procedure increased their therapeutic benefits on diabetic mice, inducing improvements in peripheral nerve function, intraepidermal nerve fiber density, remyelination, and blood flow. After intravenous injection, miR-146a-BM-exosomes levels were elevated in the sciatic nerve, and co-staining analysis indicated that exosomes were absorbed by macrophages. Exosomes facilitated M2 polarization and suppressed the release of inflammatory cytokines, which were increased due to DM [53].

## 4. Mesenchymal Stromal Cells in Diabetic Neuropathic Pain 

**Figure 1:**
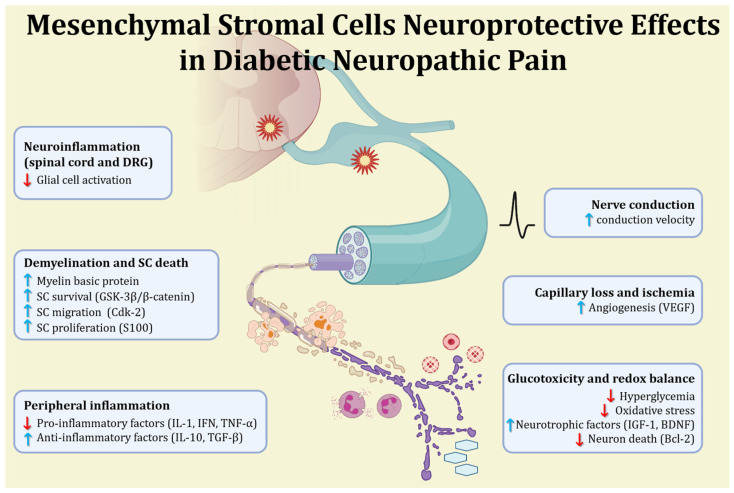
Multiple mechanisms involved in the neuroprotective action of MSCs in painful DN.

Current treatments for neuropathic pain induced by DM, which include opioid analgesics, antidepressants, and anticonvulsants, produce pain relief to only a limited number of diabetic patients [54,55]. Pain-related behaviors detected in animal models closely resemble the symptoms observed in patients with painful DN. Diabetic rodents show elevated blood levels of IL-6, cyclooxygenase-2, inducible nitric oxide synthase (iNOS), VEGF, and TNF-α. Intrathecal or intravenous administration of MSCs has been reported to produce analgesic effects on neuropathic pain of diverse causes because of their anti-inflammatory and immunomodulatory activities [56,57]. MSCs relieve pain by reducing the levels of synaptophysin and TNF-α, particularly in spinal cord and dorsal root ganglia [58]. Transplantation of autologous BM-MSCs reduced hyperalgesia and allodynia in diabetic rats, along with a recovery of nerve function [59]. The improvement in DN-associated neuropathic pain following MSC transplantation occur through rapid immune modulation in the peripheral system, thus resulting in an increased release of anti-inflammatory factors and interfering with immune cell recruitment and activation (Figure 1) [60]. Additionally, MSCs also secrete cytokines that promote cell regeneration and neovascularization, potentially improving blood flow and nerve function in DM [61]. The reduction in neuroinflammation induced by MSCs is likely a consequence of their cytokine secretion, facilitating their migration toward damaged tissue. Moreover, the decrease in serum IL-10 may interfere with inflammation and reduce sensory hypersensitivity [62]. 

Moreover, intravenous administration of CM from AT-MSCs reduced thermal hyperalgesia and recovered mechanical sensitivity in STZ-DM and diabetic *db*/*db* mice [63,64]. These benefits were associated with a decrease in T-lymphocyte and macrophage infiltration and a reduction in the expression of pro-inflammatory cytokines [63]. Both AT-MSCs and their secretome reduced pro-inflammatory factors (IL-1β, IL-6, and TNF-α) in the dorsal root ganglia, sciatic nerve, and spinal cord, suggesting a reduction in neuroinflammation. The secretome also upregulated neurotrophic factors, such as BDNF, NGF, NT3, and glial-cell-line-derived neurotrophic factor (GDNF); angiogenic factors, such as VEGF, basic fibroblast growth factor (bFGF), and platelet-derived growth factor (PDGF); and anti-inflammatory cytokines, particularly IL-10 and TGF-β [64]. The nerve damage and microangiopathy of DN are associated with endothelial dysfunction and impaired angiogenesis and result in local ischemia, but nerve blood flow was increased following MSC administration.

Neuroinflammation plays a significant role in pain induced by peripheral nerve damage as activated microglia is involved in different signaling cascades that increase neuronal excitability and modulate synaptic plasticity. The upregulation of pro-inflammatory factors by these cells aggravates hyperalgesia and allodynia, and their inhibition by MSC transplantation is likely due to a decrease in chemokine ligand 7 (CCL7) levels, a key factor involved in neuropathic pain pathogenesis [65]. 

The secretion of endocannabinoids by MSCs may also contribute to their analgesic effect. Activation of cannabinoid receptor CB1, expressed in peripheral nociceptors, increases the formation of VEGF and TGF-β, while CB2 on microglia modulates the progression of neuropathic pain [66]. Increased glial activation in the spinal cord sustains an inflammatory milieu and facilitates glutamatergic signaling, processes attributed to microglia and astroglia, respectively. Modulation of microglial CB2 receptors downregulates purinergic receptors in neuropathic pain, with s concomitant decrease in signaling through NF-κB and p38 MAPK, thus promoting a reduction in NO synthesis [67,68,69]. Furthermore, this activation also increases astrocyte resistance to ROS. The improvement of glutamate removal by astrocytes could also ameliorate the cognitive impairment found in DM. 

Another promising therapeutic alternative to treat diseases is the use of extracellular vesicles secreted by MSCs, particularly exosomes containing transmembrane proteins, mRNA, microRNA, and DNA [70,71]. In several animal models of chronic pain, a component of UC-MSCs exosomes, miR-146a-5p, decreases neuroinflammation in the spinal cord, reducing inflammatory markers and leading to pain relief. Chronic pain induced by nerve injury in rats was reduced after intrathecal infusion of MSC exosomes [72]. 

## 5. Clinical Translation of Mesenchymal Stromal Cells Therapy

Treatment of painful DN typically follows guidelines supported by randomized controlled trials, which recommend amitriptyline, duloxetine, pregabalin, and gabapentin as first-line therapies [73]. However, the outcome of monotherapy is not adequate to pain relief, and combination treatments are not approved by regulatory agencies due to inconsistent evidence. Effective pain management is crucial as patients with DN neuropathic pain are at increased risk of vascular events and mortality [74]. Given the multifactorial nature of DN pathogenesis, alternative therapies targeting mitochondrial dysfunction, oxidative stress, and neuroinflammation are under investigation. Considering that the current therapy of painful DN does not benefit all diabetic patients, MSC transplantation offers promising immunomodulatory [75] and paracrine [76] regulatory functions. 

Half of clinical trials (Phase I or II) investigating MSC-based therapy are related to diseases of bone, the brain, and the immune system [77]. However, conflicting clinical evidence regarding MSC treatment in diabetic patients arises due to the varying need for single- versus multiple-administration schemes to reduce insulin requirements [78]. Additionally, treatment protocols may not be ideal for all stages of DN given the different structural or functional changes and different nerve fibers affected (sensory, autonomic, and motor). In some cases, MSCs reversed the insulin resistance but did not improve the immune dysfunction. The efficacy of MSCs in reducing inflammatory factors and increasing anti-inflammatory cytokines to modulate the progression of DM was also observed in diabetic children [79]. However, clinical trials demonstrating the modulation of immune response by MSCs in diabetic patients have not examined the presence of complications such as neuropathic pain. The primary focus of these trials has been to explore the potential of MSCs to restore health and function of pancreas [59]. However, there is no clear evidence confirming the development of mature, functioning pancreatic β-cells. 

Recently, human embryonic stem cells (hESCs) and induced pluripotent stem cells (iPSCs) have been evaluated in diabetic patients, showing potential to produce functional pancreatic β-cells, possibly through paracrine effects [59]. Long-term DM often leads to delayed wound healing, with severe consequences such as infection and pain. MSCs have demonstrated the ability to reduce pain and promote healing in diabetic patients with ischemic ulcers by decreasing fibrosis, modulating immune cell activity, and promoting angiogenesis. Thus, MSCs regulate the inflammatory conditions, promoting vascularization and anti-oxidant activity, y, leading to healing of the diabetic ulcer and reducing the rate of amputations. Neuronal recovery following MSC therapy is attributed to the formation of BDNF, NGF, and GDNF [80,81]. Despite their therapeutic potential, this alternative is not yet used in clinical practice for the treatment of painful DN. Despite numerous clinical studies demonstrating the safety of MSCs, their clinical application for pain treatment remains limited due to challenges such as reduced migration of transplanted cells to tissues, low cell survival in inflammatory conditions, and risk of pulmonary thrombosis [82]. Therefore, no clinical trial is in progress or registered on ClinicalTrials.gov for the investigation of MSC cell or exosome therapy in DN neuropathic pain patients.

Advances in clinical use pertain to the case of foot ulcers in diabetic patients, who can benefit from wound healing as a result of better vascularization and modulation of the immune system. MSCs can interfere with each stage of the wound healing process because of the effects of angiogenesis and tissue remodeling [83]. The use of advanced technology is necessary to improve the effectiveness of transplantation. Recently, MSC-secreted exosomes (extracellular vesicles) have been of interest to researchers because of their composition of proteins, mRNA, and microRNA, regulating gene expression and activity via intercellular communication [84]. Exosomes are nanoscale lipid bilayer vesicles which cross biological barriers and have a low probability of producing embolism. Since MSCs have affinity to tumor sites, MSC-derived exosomes can modulate the tumor domain through the facilitation of anticancer drug delivery with low toxicity. Thus, MSC-derived exosomes have the potential to increase the therapeutic sensitivity of anticancer agents [85]. Few clinical trials of MSC exosomes in diabetic patients are available to provide information regarding their safety and therapeutic effects. No clinical trial on the MSC treatment of painful DN supports ideal therapeutic protocol or safety. No study has yet conducted an analysis on the side effects produced by MSC transplantation for the management of diabetic neuropathic pain. 

## 6. Conclusions

The complex pathogenesis of neuropathic pain has focused attention on not only the multidisciplinary care of diabetic patients but also on the introduction of multi-target therapies. There is no specific treatment for painful DN, and new approaches should consider the prevention of oxidative stress, the reduction of inflammatory molecules, and interference with metabolic disturbance. In animal models, the paracrine effects of neurotrophic and angiogenic factors induced by MSCs can modulate the myelination in peripheral nerves and the axonal regeneration, associated with recovered structure and function. Patients with DN commonly have severe pain, which can lead to depression, anxiety, and sleep disorders [86]; therefore, it is a clinical challenge to prevent or treat this condition. MSCs are promising alternatives, providing disease modification through the reversion of nerve damage through the secretion of trophic (regenerative) factors, vascular endothelial growth factor, and nitric oxide [87]. Current research also highlights the therapeutic potential of MSC exosomes owing to the reduced risk of tumor formation and embolism during transplantation. Moreover, thorough investigations of the pharmacological properties of MSC and their exosomes still need to be conducted in order to determine the optimal cell source and mode of priming, the route of administration, and the therapeutic regimen. However, further multicenter investigation using MSC transplantation to prevent or treat neuropathic pain in diabetic patients is necessary to ensure safe and adequate pharmacological effects.

## Data Availability

Not applicable.
